# Incidence and clinical features of the incidentally found vascular stump thrombus during routine follow up after oncologic lung surgery

**DOI:** 10.1371/journal.pone.0185140

**Published:** 2017-09-27

**Authors:** Mi Hyoung Moon, Kyongmin Sarah Beck, Young Kyu Moon, Jae Kil Park, Sook Whan Sung

**Affiliations:** 1 Department of Thoracic and Cardiovascular Surgery, Seoul St. Mary’s Hospital, Catholic University of Korea, Seoul, Republic of Korea; 2 Department of Radiology, Seoul St. Mary’s Hospital, Catholic University of Korea, Seoul, Republic of Korea; Baylor College of Medicine, UNITED STATES

## Abstract

**Objectives:**

We aimed to evaluate the incidence and clinical features of vascular stump thrombus after oncologic lung surgery.

**Methods:**

A retrospective analysis of records from our institutional database dated between 2009 and 2016 was performed. Data regarding demographics, clinical presentation, medication use, operative findings, pathology, and radiologic findings were retrieved.

**Results:**

The study cohort consisted of 648 oncologic surgeries for primary lung cancer. The incidence of thrombus in the entire population was 5.7% (37/648). Most thrombi were incidentally found on follow-up chest computed tomography scans. Univariate Cox proportional hazard analysis showed that age (p = 0.02), adjuvant therapy (p <0.001), neoadjuvant therapy (p = 0.04), left-sided surgery (p = 0.02), complex surgery greater than simple lobectomy or segmentectomy (p <0.001), advanced stages (p <0.001), non-adenocarcinoma (p = 0.003), and thoracotomy approach (p = 0.009) were associated with an increased risk of vascular stump thrombus. There were no embolic events in our cohort, except for a case of pulmonary thromboembolism. During follow-up, 43.2% (16/37) of thrombi had completely resolved, 48.6% (18/37) showed partial regression and stabilization, and 8.1% (3/37) had progressed.

**Conclusions:**

The incidence of vascular stump thrombus in our study was not negligible. The clinical course of stump thrombus appears to be benign in most cases. Anticoagulation may be used with caution based on an individual basis depending on each patient’s risk factors.

## Introduction

Venous thromboembolism (VTE), including pulmonary thromboembolism (PTE) and deep venous thrombosis (DVT), is a potentially fatal complication. The incidence of VTE after oncologic lung surgery has been reported to be 5.3 to 7.4%, and the peak incidence is within the first month after surgery (2%) [1, 2].

Since the first case of pulmonary vein stump thrombus extending into the left atrium was reported by Seki et al. in 1989, several subsequent case reports have also identified “stump thrombosis” in either the pulmonary vein or artery stump [3, 4]. In the past, vascular stump thrombus had been thought to exist as a part of PTE [5]. However, this entity is now regarded as a different postsurgical thrombosis [6, 7].

It is important to distinguish vascular stump thrombus, recurrence, and embolization from DVT because the natural history, treatment, and prognosis are likely to be different. Vascular stump thrombus can be asymptomatic and may be identified as an incidental finding on follow-up computed tomography (CT) scans, but can also cause systemic infarction to the brain or kidney [3, 4, 8, 9]. Most reports examining stump thrombus focus on systemic infarction or embolization, so the exact incidence or natural course of this phenomenon is unclear. Moreover, the indications for pharmacologic prophylaxis or treatment also remain unclear. The purpose of this study is to assess the incidence, clinical features, and possible risk factors of vascular stump thrombus after oncologic lung surgery with curative intent.

## Materials and methods

### Study population and data collection

A retrospective review of the prospectively collected surgical database was performed to identify patients who underwent primary lung cancer surgery with curative intent from May, 2009 to March, 2016. We excluded patients who had: (1) a history of DVT or PTE prior to surgery, (2) an inferior vena cava filter, (3) a history of other malignancy, or (4) no follow-up contrast-enhanced CT. We also excluded patients with postoperative PTE, benign pathology, or who underwent surgery with palliative or noncurative intent. This study was approved by the Institutional Review Board of the Seoul St. Mary’s Hospital (approval no. KC16RISI0907). The requirement of informed consent from individual patients was waived since this study was a retrospective review of a database and medical records.

### Preoperative evaluation and postoperative follow up

The preoperative staging evaluation included a detailed history taking and physical examination, chest x-ray, standard laboratory tests, flexible bronchoscopy, contrast-enhanced chest CT scan, whole body fluorodeoxyglucose positron emission tomography /computed tomography (FDG PET/CT), and whole body bone scan. Transthoracic echocardiography was performed for patients with a history of ischemic heart disease or valvular heart disease. The decision on treatment modalities was made by the interdisciplinary tumor board that has been in place at our institution since 2005, consisting of thoracic surgeons, medical oncologists, radiologic oncologists, and radiologists. All patients were registered to the departmental Lung Cancer Database. Postoperatively, the operative and pathologic findings were discussed again at the tumor board and further treatment was decided upon when indicated.

A postoperative follow-up visit occurred within one week of hospital discharge. The first contrast-enhanced chest CT scan was performed within three months after surgery. If the patient was scheduled to receive an adjuvant therapy, the first follow-up chest CT scan was performed prior to the initiation of the adjuvant therapy. The subsequent postoperative follow-up schedule consisted of outpatient clinic visits every 3–6 months until the fifth year, then every 6 or 12 months depending on the patient’s wishes. CT scans were performed within 3 months post-operatively, as noted above, and then every 6 months for the first five years. This schedule could be modified if the patient received adjuvant therapy or had any respiratory signs or symptoms. After the first five years of follow-up, the contrast-enhanced chest CT scan was performed yearly. While contrast-enhanced CT was the usual modality performed during the first year, non-enhanced CT scans were performed in some patients, especially after two years without recurrence or in patients who had poor renal function.

### Postoperative management

In our institution, anti-embolism stockings are applied to all patients during surgery for DVT prophylaxis. When a patient is hospitalized, a customized program for DVT prophylaxis incorporated into our Electronic Medical Records (EMR) system classifies the patient as low, medium, or high risk of DVT development and recommends appropriate management to the physician. All the thoracic surgical patients had the anti-embolism stockings applied. For the patients who received neoadjuvant therapy, half-dose of low molecular weight heparin (0.5mg/kg) was administered postoperatively. Since all patients were routinely transferred to the general ward and began to ambulate immediately with the help of a trained nurse, routine postoperative heparin was not administered to the remaining patients. In 2009, 100mg aspirin was administered to 37 patients as a component of prophylaxis. There were no morbidities related to aspirin. We subsequently decided to administer dual antiplatelet prophylaxis (aspirin 100mg and clopidogrel 75mg), which was initiated on postoperative day 1 and continued for 1 to 3 months after surgery, depending on each patient’s compliance. Since this study was performed to investigate the incidence and clinical features of stump thrombi, we included all patients who developed vascular stump thrombus regardless of whether they received single or dual antiplatelet agents. In this study group, 37 patients received aspirin alone, and the 400 patients received dual agents.

### Identification of stump thrombus

Contrast injection was performed by a mechanical injector at a rate of 3ml/sec up to a total dose of 100ml. Based on the raw data, the axial, sagittal and coronal images were reconstructed at 3 mm thickness. The radiographic identification of stump thrombus was defined as the presence of a solitary filling defect with soft tissue attenuation confined to the pulmonary arterial or venous stump and the absence of remote thrombus. In addition, the presence of a well-defined soft tissue density located within the stump without evidence of extravascular extension and its resolution or stability on follow-up CT scan was recognized as a finding favoring vascular stump thrombus [6]. The time of onset or resolution of vascular stump thrombus cannot be dated precisely because the CT scans were obtained routinely. For convenience, we defined the onset of the stump thrombus as the first date that the thrombus was detected on CT scan, and the date of resolution as the date in which disappearance of thrombus was confirmed on CT scan.

### Statistical analysis

Collected data included patient characteristics and demographics, comorbidities, medication history, alcohol and tobacco use, tumor characteristics, and neoadjuvant and adjuvant therapies. In addition, the date of surgery, type of surgery [video assisted thoracoscopic surgery (VATS) or thoracotomy], extent of resection, duration of chest tube presence, length of total hospital stay, length of postoperative hospital stay, postoperative morbidity, and in-hospital mortality were recorded. For the analysis, the extent of resection was categorized into two groups: 1) simple lobectomy and segmentectomy was considered to be the “simple lobectomy or less” group, and 2) sleeve resection, bilobectomy, or pneumonectomy was considered to be the “more complex procedure” group. Staging was determined according to the TNM Classification of Malignant Tumors, Seventh Edition. Histopathological classification was determined according to the International Multidisciplinary Classification of Lung Adenocarcinoma [10].

Characteristics were compared between the groups with and without vascular stump thrombus. Continuous variables are presented as mean ± standard deviation, whereas categorical variables are presented as frequencies and percentages. The distributions of continuous variables were analyzed by the Student’s t-test or Mann-Whitney U test depending on the result of Shapiro-Wilk test of normality. Categorical variables were compared using the Chi-square test or the Fisher’s exact test.

To assess the possible risk factors associated with thrombus development, univariate Cox proportional hazard (PH) analysis was performed. Because the event was small in this study, we did not perform multivariate Cox PH analysis. All statistical analyses were performed using R ver. 3.3.3 [R Core Team (2013). R: A language and environment for statistical computing. R Foundation for Statistical Computing, Vienna, Austria. URL http://www.R-project.org/].

## Results

### Characteristics of patients with stump thrombus

From May, 2009 to March, 2016, a total of 1196 pulmonary resections were performed. Among them, patients with recurrent or metastatic cancers (492 patients), a previous history of PTE or DVT or the presence of an IVC filter (5 patients), postoperative PTE (33 patients), an absence of contrast-enhanced CT scans (6 patients), and those who underwent surgeries with non-curative intent (12 patients) were excluded.

The crude incidence of vascular stump thrombus was 5.7% (37/648). The baseline characteristics of the patients with or without stump thrombus are shown in Table 1. The baseline comorbidity profiles, including diabetes mellitus (p = 0.66), hypertension (p = 0.95), chronic obstructive lung disease (p > 0.99), peripheral vascular disease or ischemic heart disease (p > 0.99), previous history of cerebrovascular accident (p = 0.64), history of other cancer (p = 0.69), or habitual alcohol use (p = 0.77) did not vary between the two groups. The Charlson-Deyo score, preoperative pulmonary function, preoperative performance status (ECOG), and preoperative atrial fibrillation (p = 0.30) and other comorbidities were not statistically different between the two groups.

**Table 1:** Clinical characteristics of patients with and without vascular stump thrombus.

	Vascular stump thrombus		P value
	No (n = 611)	Yes (n = 37)	
Female sex	356 (58.3%)	25 (67.6%)	0.35
Age at operation (years)	63.9 ± 9.6	67.5 ± 8.0	0.03
Preoperative ECOG score	0.03 ± 0.19	0.05 ± 0.23	0.49
Charlson-Deyo score	3.77 ± 1.71	3.92 ± 1.52	0.51
Smoking status			0.22
Current smoker	80 (13.1%)	8 (21.6%)	
Nonsmoker	534 (86.9%)	29 (78.4%)	
Pre-op lung function			
FEV1 (l)	2.37 ± 0.57	2.33 ± 0.66	0.66
FVC (l)	3.34 ± 0.76	3.35 ± 0.86	0.92
FEV1/ FVC	71.57 ± 10.15	70.54 ± 8.65	0.26
DLCO/VA	3.56 ± 0.82	3.51 ± 0.82	0.76
Neoadjuvant therapy	60 (9.8%)	7 (18.9%)	0.09
Adjuvant therapy	211 (34.5%)	31 (83.8%)	< 0.001
Types of adjuvant therapy			< 0.001
None	400 (65.5%)	5 (13.5%)	
Chemotherapy	143 (23.4%)	21 (56.8%)	
Radiotherapy	10 (1.6%)	2 (5.4%)	
CCRT	58 (9.5%)	9 (24.3%)	
Histology			0.007
Adenocarcinoma	434 (71.0%)	18 (48.6%)	
Other	177 (29.0%)	19 (51.4%)	
Stage			< 0.001
I	409 (66.9%)	9 (24.3%)	
II	102 (16.7%)	13 (35.1%)	
III	80 (13.1%)	14 (37.8%)	
IV	20 (3.3%)	1 (2.7%)	
Left-sided lesions	239 (39.1%)	22 (59.5%)	0.02
Tumor location			<0.001
RUL	201 (32.9%)	1 (2.7%)	
RML	57 (9.3%)	4 (10.8%)	
RLL	114 (18.7%)	10 (27.0%)	
LUL	136 (22.3%)	12 (32.4%)	
LLL	103 (16.9%)	10 (27.0%)	
Operation type			0.001
VATS	505 (82.7%)	22 (59.5%)	
Open thoracotomy	106 (17.3%)	15 (40.5%)	
Resection			< 0.001
Lobectomy or less	572 (93.6%)	25 (67.6%)	
Complex procedure	39 (6.4%)	12 (32.4%)	
Anesthesia duration (min)	220.4 ± 70.20	254.11 ± 64.20	< 0.001
Operative time (min)	170.82 ± 68.42	203.14 ± 65.61	0.001
Lymph node dissection			0.02
MLND	487 (79.7%)	36 (97.3%)	
MLS	48 (7.9%)	0 (0.0%)	
None	76 (12.4%)	1 (2.7%)	
Postoperative antiplatelets	420 (68.6%)	17 (45.9%)	0.003
Discharge after operation (days)	7.4 ± 6.4	9.3 ± 8.2	0.02
Chest tube indwelling time (days)	5.8 ± 9.2	7.1 ± 9.2	0.05
Postoperative morbidity	129 (21.1%)	9 (24.3%)	0.80
BPF/Empyema	1 (0.2%)	2 (5.4%)	<0.001
Persistent air leakage	79 (12.9%)	4 (10.8%)	> 0.99
Postoperative atrial fibrillation	15 (2.5%)	1 (2.7%)	0.61
Readmission	15 (2.5%)	2 (5.4%)	0.25

BPF: bronchopulmonary fistula, CCRT: concurrent chemoradiotherapy, DLCO: diffusion capacity for carbon monoxide, ECOG: Eastern Cooperative Oncology Group, FEV1: forced expiratory volume 1 second, FVC: forced vital capacity, LLL: left lower lobe, LUL: left upper lobe, MLND: mediastinal lymph node dissection, MLS: mediastinal lymph node sampling, RML: right middle lobe, RLL: right lower lobe, RUL: right upper lobe, VA: alveolar volume, VATS: video-assisted thoracoscopic surgery.

However, patients with stump thrombus tended to be slightly older, more likely to have received adjuvant therapy, have stage II or III, have left sided lesions, have undergone a thoracotomy approach, and have received more than a simple lobectomy. There was no significant difference in the usage of postoperative coagulant (p = 0.70), postoperative oral anticoagulant (p > 0.99), postoperative heparin (p = 0.83) between the two groups. Postoperatively, patients with stump thrombus showed slightly longer hospital stays, but the development of postoperative atrial fibrillation on the first postoperative day was not statistically different.

### CT findings and characteristics of stump thrombi

A total of thirty-seven patients developed stump thrombus; 25 involved arterial stumps and 12 involved venous stumps. The median time interval from surgery to the first detection of all stump thrombi, arterial thrombi, and venous thrombi were 28 days (range: 20 to 341), 26 days (range: 20 to 302), and 30 days (range: 22 to 341), respectively.

**Fig 1:**
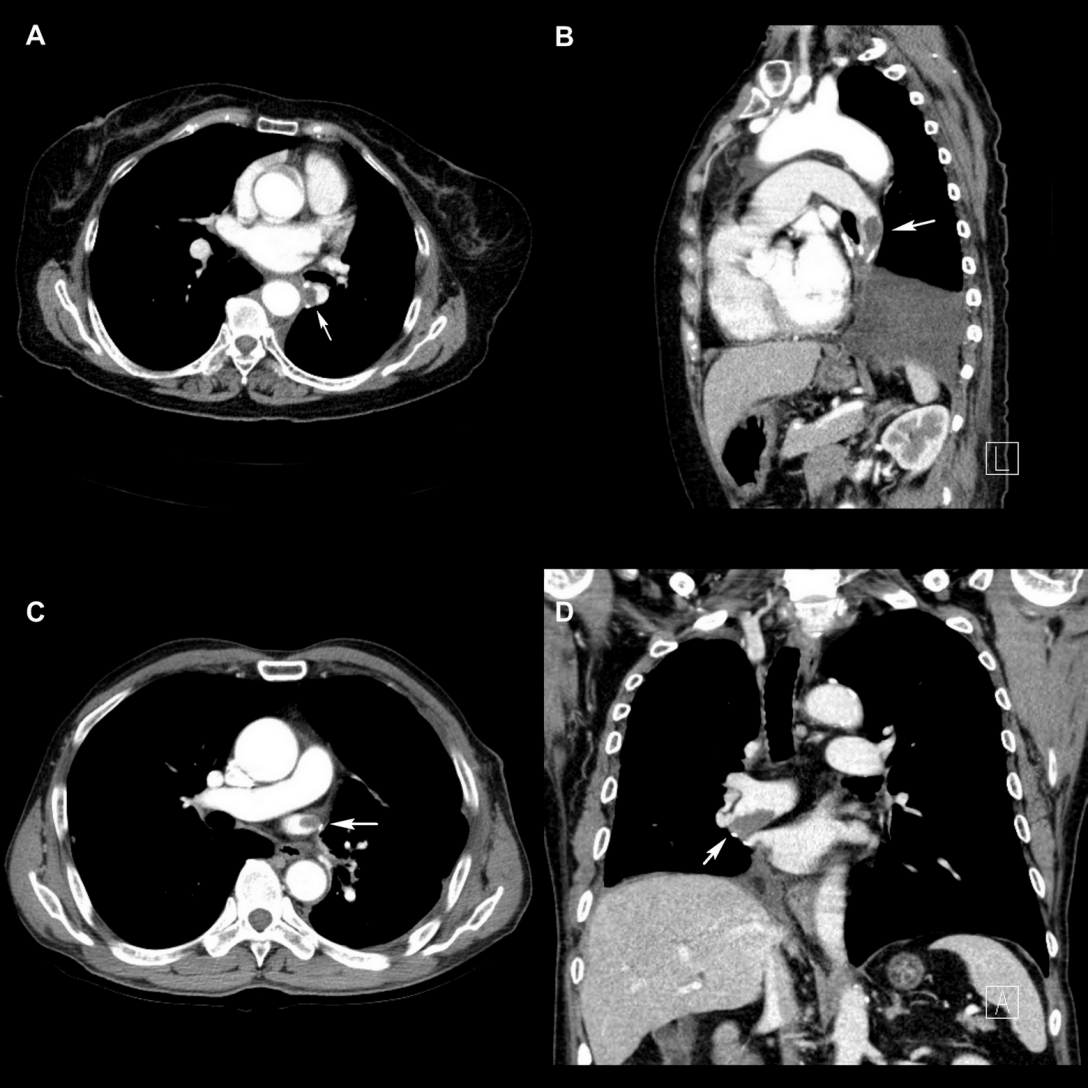
Various shapes and positions of vascular stump thrombi. (A) Axial image of a round and convex pulmonary artery thrombus after VATS left lower lobe lobectomy. (B) Sagittal image from the same patient, which shows the thrombus better than the axial image. (C) A small round thrombus is identified at the left superior pulmonary vein stump after left upper lobe lobectomy. (D) A large irregular thrombus identified at the right inferior pulmonary vein stump.

The shape and length of the thrombi were variable (Fig 1). The mean length of all stump thrombi, defined as the longest diameter measured in any of the axial, coronal, and sagittal planes, was 15.5 mm. The arterial thrombi were longer than the venous thrombi (p = 0.04). The mean lengths of arterial thrombi and venous thrombi were 16.8 mm and 12.8 mm, respectively. All stump thrombi showed soft tissue attenuation, measuring 44.3 Hounsfield unit (HU) in average. Though marginally significant (p = 0.06), arterial thrombi (40.4 ± 14.6 HU) tended to show slightly lower attenuation than venous thrombi (52.3 ± 18.2 HU). The majority of the stump thrombi (32 total; 21 arterial and 11 venous) were convex in shape, while 5 stump thrombi (4 arterial and 1 venous) were concave. Stump thrombi were more frequently found in the left-sided stumps (n = 22) than in the right-sided stump (n = 15) (p <0.001), with all 12 of the venous thrombi having been found exclusively in the left side.

### Clinical features and natural course

All thrombi were found incidentally on the scheduled CT scans. None of the patients complained of leg swelling or new dyspnea. Although the detection time was dependent on the timing of scheduled CT scans, most of the thrombi were found early and 64.9% (24/37) of thrombi were found within one month after surgery. Among 37 patients, a total of five patients received anticoagulant treatment consisting of either low molecular weight heparin (LMWH) followed by an oral anticoagulant (warfarin or rivaroxaban) or oral anticoagulants only. The duration of oral anticoagulant use was 6 months to 1 year. One of these treated patients, the 69-year old female who developed a right lower lobar pulmonary artery stump thrombus after right lower lobe lobectomy, was initially observed without pharmacologic intervention until LMWH and oral anticoagulation were ultimately initiated when the growing thrombus was identified on follow-up CT scans 6 months later. Seventeen patients were taking antiplatelet agents when the thrombi were found. In four patients, the antiplatelet agents were stopped and anticoagulant therapy was started as stated above, while the antiplatelet medication was continued for the remaining patients.

**Fig 2:**
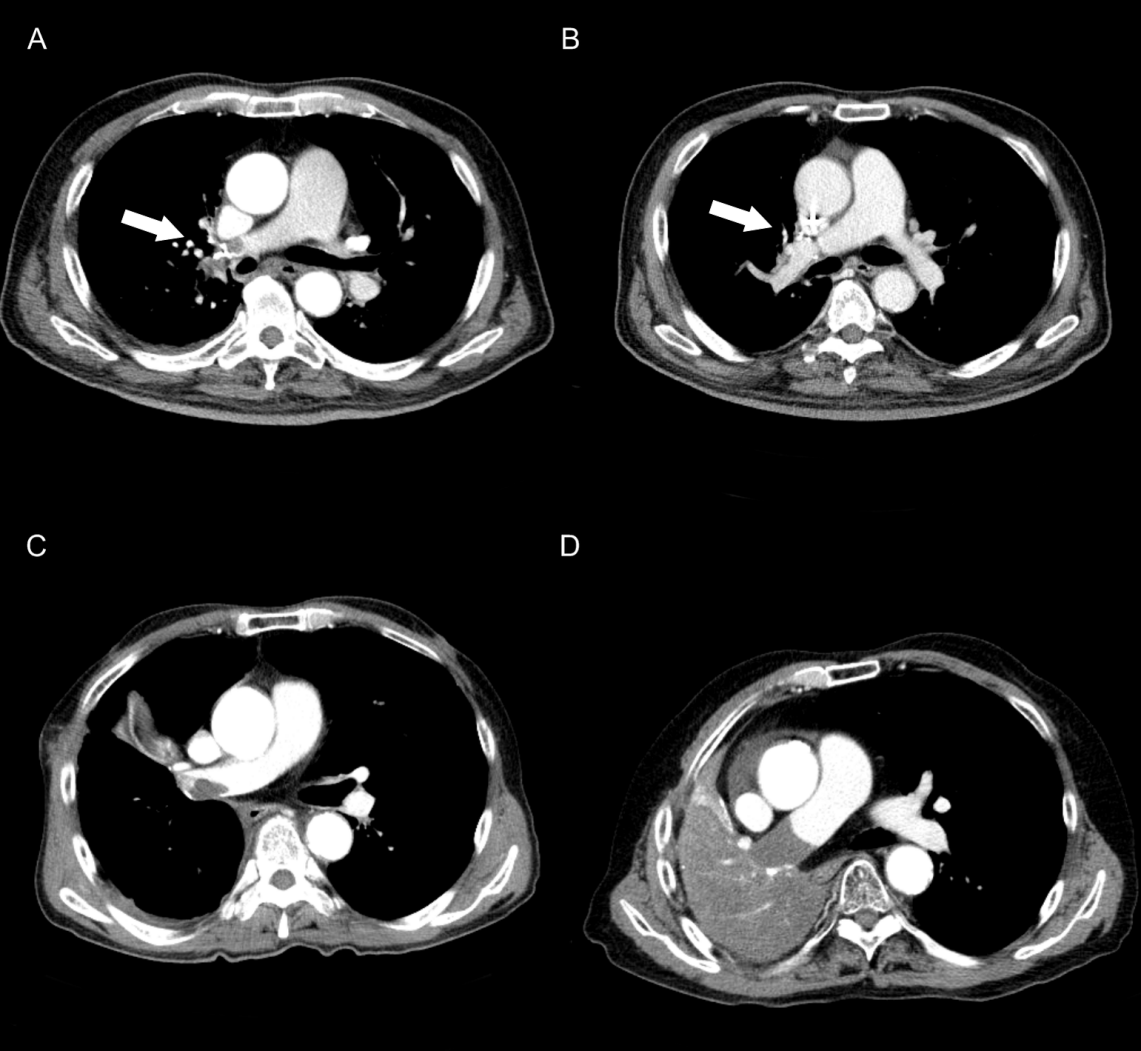
Various courses of vascular stump thrombi on follow-up CT scans. (A) A 77-year-old female status post VATS RUL lobectomy developed pulmonary arterial vascular stump thrombi on the first follow-up CT scan (26 days after surgery). (B) At the second follow-up CT scan, the thrombus had resolved spontaneously (the white arrow indicates the vascular stump). (C) A 72-year-old female status post right middle and lower lobe bilobectomy developed a pulmonary arterial vascular stump thrombus, which was detected 22 days after surgery. (D) The pulmonary arterial stump thrombus first shown as C had grown to completely obstruct the right main pulmonary artery.

The clinical courses of the stump thrombi were generally benign. Most of the stump thrombi showed either complete resolution or partial regression (Fig 2A and 2B) upon repeat imaging. Of 37 stump thrombi, 16 (12 arterial and 4 venous) showed complete resolution on CT after a median of 106 days (range: 58 to 687 days), and 18 (12 arterial and 6 venous) showed shrinkage of both the thrombi and the vessels, with diminished thrombi still remaining within the shrunken vessel lumens (Table 2). Of the remaining three stump thrombi, two thrombi stabilized after increasing in size without regression, and one showed progression. The patient whose thrombus showed progression on follow up CT scan was started on anticoagulation, but the patient died from respiratory failure and progression of the lung cancer (Fig 2C and 2D). Another patient developed PTE without evidence of DVT 4 years after complete regression of left upper lobar pulmonary vein stump thrombi. The patient complained of dyspnea, and CT scan showed small PTE. There were neither embolic events nor mortality caused by directly by stump thrombi in our patients.

**Table 2:** Radiologic features of stump thrombi.

	Arterial stump thrombi (n = 25)	Venous stump thrombi (n = 12)	Total (n = 37)
Location of filling defect			
Right-sided stump	15	0	15
Left-sided stump	10	12	22
Thrombus characteristics			
Convex shape	21	11	32
Concave shape	4	1	5
Length* (mm)	16.8 ± 6.9	12.8 ± 4.7	15.5 ± 6.5
Attenuation* (Hounsefield unit)	40.4 ± 14.6	52.3 ± 18.2	44.3 ± 16.6
First appearance following surgery^†^	26 (20–302)	30 (22–341)	28 (20–341)
Findings at follow-up			
Complete resolution	12	4	16
Partial regression	12	6	18
Progression and/or stabilization	1	2	3
Time to complete resolution^†^	99 (58–597)	251 (100–687)	106 (58–687)

*: mean ± standard deviation

^†^: days, median (ranges)

### Factors associated with stump thrombus

To assess the possible risk factors for vascular stump thrombus, univariate Cox PH analysis was done (Table 3). Due to the small number of events, we did not perform multivariate analysis. First, either Charson-Deyo score, ischemic heart disease (p = 0.59), preoperative atrial fibrillation (p = 0.39), or preoperative ECOG (p = 0.39) were not significant risk factors for the thrombus development. Elderly age, advanced stage, and neoadjuvant therapy were the risk factors identified for the development of stump thrombus. Factors reflecting more extensive surgery (thoracotomy approach rather than VATS approach, complex surgeries rather than simple lobectomy, longer operative time) were also associated with higher risk. There were 1 completion pneumonectomy (left sided) and 3 pneumonectomies (2 left sided, 1 right sided), and thrombi were found in the completion pneumonectomy patient and one right pneumonectomy patient. Postoperative procoagulant use (p = 0.96), postoperative prophylactic LMWH use (p = 0.44), or newly developed postoperative atrial fibrillation (p = 0.88) did not increase the risk. Interestingly, the use of antiplatelet agents seemed to reduce the risk of vascular stump thrombus postoperatively (p = 0.02).

**Table 3:** Univariable Cox proportional hazard regression of selected risk factors for vascular stump thrombus development.

	HR	95% CI	P value
Sex: Female	1.523	0.765–3.032	0.23
Age at operation	1.051	1.012–1.090	0.009
Charlson-Deyo score	1.062	0.885–1.274	0.52
Neoadjuvant therapy	2.441	1.067–5.586	0.04
Adjuvant therapy	10.11	4.206–24.29	< 0.001
Stage	1.977	1.469–2.662	< 0.001
Non-adenocarcinoma	2.642	1.385–5.038	0.003
Laterality: Left sided	2.201	1.141–4.244	0.02
VATS vs open			
VATS	1		
Thoracotomy	2.458	1.248–4.843	0.009
Resection extent			
Simple lobectomy or less	1		
More complex procedure	6.317	3.155–12.65	< 0.001
Anesthesia time (min)	1.005	1.002–1.009	0.005
Operation time (min)	1.005	1.001–1.009	0.008
Hospital stay after operation	1.030	1.000–1.061	0.05
Antiplatelet medication	0.459	0.237–0.890	0.02

Development of any postoperative morbidities (p >0.99), prolonged air leak (p = 0.96), or postoperative acute lung injury (p = 0.09) did not increase the risk. However, development of postoperative bronchopleural fistula or empyema increased the risk (p <0.001).

## Discussion

Incidental emboli in the pulmonary vasculature following oncologic lung surgery have been reported to occur in many as 5% [7]. Previously, the vascular stump thrombi were thought as the part of PTE; however, it is now regarded as a different postsurgical thrombosis [11]. The exact incidence and clinical implications of this phenomenon have only been documented sporadically. The incidence was previously reported to be 3.54% after lobectomy, and 12% after pneumonectomy [6, 8]. In our study, the overall incidence was 5.71%, 1.85% (12/684) for vein thrombus, and 3.85% (25/684) for arterial thrombus. We cautiously speculate that our incidence reflects the actual population incidence since nearly all patients in our cohort underwent postoperative CT scan.

The exact etiology or pathogenesis of the development of either arterial or venous stump thrombus is unclear. Since Virchow’s triad has been suggested as an underlying etiology of stump thrombus, we can hypothesize three mechanisms: endothelial injury during surgery, blood flow stasis at the end pouch of the vascular stump, and hypercoagulability. Elevated plasma fibrinogen levels and peripheral blood platelet counts have been reported in patients without a past history of thrombosis-inducing activity before pulmonary resection [12]. Additionally, slow blood flow has been observed by Doppler ultrasound, especially in the left superior pulmonary vein, which is usually longer than other vascular stumps owing to its anatomic position [13]. Anatomy might be another predisposing factor. In this study, left-sided thrombi were more common than right-sided ones. After lobectomy, left-sided vascular stump is longer than the right-sided ones. This long vascular stump “pouch” can contribute to blood flow turbulence [14]. After pneumonectomy, the relationship between the operation side and the predisposing side for thrombi is confusing, since multiple reports describe differently [6, 15]. Complex procedures requiring more procedural time such as bilobectomy, completion lobectomy, or completion pneumonectomy were associated with an increased risk of stump thrombus in our study. Complex and long procedures reflect more vascular manipulation with a subsequent increased chance of endothelial injury.

In the literature, there are reported to be two types of thrombi [6]. The first type is high-risk thrombi that may embolize or grow with time. The floating or convex types seem to be more acute and have higher risk of embolism than the concave types. Some researchers recommend the initiation of anticoagulation for this type of thrombi [16]. The other type is a concave, benign thrombus with low risk of embolism. This type of thrombus tends to resolve spontaneously or remain stable.

There is no consensus regarding the management of vascular stump thrombi. Several studies reported the systemic heparinization or anticoagulation[9, 13, 17, 18]. We cannot prematurely conclude about the use of anticoagulation in this situation, however, a subset of patients may benefit from it. In the setting of convex shaped thrombus, history of pneumonectomy or enlarging thrombus, systematic anticoagulation may be considered after weighing the risks and benefits

The NCCN guidelines recommend that all surgical oncologic patients should receive pharmacologic prophylaxis with either unfractionated or low molecular weight heparin during their hospitalization [19]. There is no recommendation after discharge, despite the evidences of persistent thrombogenic activity among these patients [20]. Currently, it remains unclear whether prophylactic or therapeutic intervention of vascular stump thrombi after oncologic lung surgery is indicated. When the stump thrombus is found, close observation with serial CTs is needed, and the decision to initiate anticoagulation should be made on an individual basis.

### Limitation

This study has important limitations stemming from the small population, retrospective design, and single center experience. Further studies are required to validate the results of this study among a larger population in a prospective randomized study.

## Conclusion

In conclusion, vascular stump thrombus occurs early in the postoperative period with incidence of around 5.7%. Its clinical course is generally benign, but progression or embolism may occur in some cases causing clinically significant effects. Since it is not an uncommon postoperative incidental finding, close observation and follow-up are warranted in the majority of patients.

## Supporting information

S1 File Dataest. (CSV)

## References

[1] Tesselaar MET, Osanto S. Risk of venous thromboembolism in lung cancer. Curr Opin Pulm Med 2007; 13:362–7. doi: 10.1097/MCP.0b013e328209413c 17940477 10.1097/MCP.0b013e328209413c

[2] Yang Y, Zhou Z, Niu XM, Li ZM, Chen ZW, Jian H, et al Clinical analysis of postoperative venous thromboembolim risk factors in lung cancer patients. J Surg Oncol 2012;106:736–41. doi: 10.1002/jso.23190 22711667 10.1002/jso.23190

[3] Ohtaka K, Hida Y, Kaga K, Iimura Y, Shiina N, Muto J, et al Pulmonary vein thrombosis after video-assisted thoracoscopic left upper lobectomy. J Thorac Cardiovasc Surg 2012;143(1):e3–5. doi: 10.1016/j.jtcvs.2011.09.025 22014720 10.1016/j.jtcvs.2011.09.025

[4] Hideo I, Yuichiro O, Hidetaka N, Seiji S. Thrombus formation in the pulmonary vein stump after left upper lobectomy: a report of four cases. Ann Thorac Cardiovasc Surg 2014;20 Suppl:613–6. doi: 10.5761/atcs.cr.13-00079 23774615 10.5761/atcs.cr.13-00079

[5] Chuang TH, Dooling JA, Connolly JM, Shefts LM. Pulmonary embolization from vascular stump thrombosis following pneumonectomy. Ann Thorac Surg 1966;2(3): 290–8. 5333596 10.1016/s0003-4975(10)66581-2

[6] Kim SY, Seo JB, Chae EJ, Do KH, Lee JS, Song JW, et al Filling defect in a pulmonary arterial stump on CT after pneumonectomy: radiologic and clinical significance. Am J Roentgenol 2005;185;985–8. doi: 10.2214/AJR.04.1515 16177420 10.2214/AJR.04.1515

[7] Gosselin MV, Rubin GD, Leung AN, Huang J, Rizk NW. Unsuspected pulmonary embolism: prospective detection on routine helical CT scans. Radiology 1998;208(1): 209–15. doi: 10.1148/radiology.208.1.9646815 9646815 10.1148/radiology.208.1.9646815

[8] Ohtaka K, Hida Y, Kaga K, Kato T, Muto J, Nakada-Kubota R, et al Thrombosis in the pulmonary vein stump after left upper lobectomy as a possible cause of cerebral infarction. Ann Thorac Surg 2013;95(1): 1924–9. doi: 10.1016/j.athoracsur.2013.03.005 23622699 10.1016/j.athoracsur.2013.03.005

[9] Barbetakis N, Asteriou C, Kleontas A. Post-lobectomy pulmonary artery stump thrombosis: How dangerous is it? Ann Thorac Surg 2011;91:e44 doi: 10.1016/j.athoracsur.2010.12.037 21352970 10.1016/j.athoracsur.2010.12.037

[10] Travis WD, Brambilia E, noguchi M, Nicholson AG, Geisinger K, Yatabe Y, et al International Association for the Study of Lung Cancer/American Thoracic Society/European Respiratory Society: International Multidisciplinary Classification of Lung Adenocarcinoma: an exexutive summary. Proc Am Thorac Soc 2011;8(5): 381–5. doi: 10.1513/pats.201107-042ST 21926387 10.1513/pats.201107-042ST

[11] Winston CB, Wechsler RJ, Salazar AM, Kurtz AB, Spirn PW. Incidental pulmonary emboli detected at helical CT: Effect on patient care. Radiology 1996;201:23–7. doi: 10.1148/radiology.201.1.8816515 8816515 10.1148/radiology.201.1.8816515

[12] Ichinose Y, Hara N, Ohta M, Hayashi S, Yahawa K. Appearance of thrombosis-inducing activity in the plasma of patients undergoing pulmonary resection. Chest 1991;100(3):693–7. 1889258 10.1378/chest.100.3.693

[13] Ohtaka K, Takahashi Y, Uemura S, Shoji Y, Hayama S, Ichimura T, et al Blood stasis may cause thrombosis in the left superior pulmonary vein stump after left upper lobectomy. J Cardiothorac Surg 2014;9:159–65. doi: 10.1186/s13019-014-0159-8 25231061 10.1186/s13019-014-0159-8 PMC4177051

[14] Ohtaka K, Hida Y, Kaga Y, Takahashi Y, Kawase H, Hayama S, et al Left upper lobectomy can be a risk factor for thrombosis in the pulmonary vein stump. J Cardiothorac Surg 2014;9:5 doi: 10.1186/1749-8090-9-5 24393449 10.1186/1749-8090-9-5 PMC3892104

[15] Kwek BH, Wittram C. Postpneumonectomy pulmonary artery stump thrombosis: CT features and imaging follow up. Radiology 2005;237(1):338–41.doi: 10.1148/radiol.2371041686 16126932 10.1148/radiol.2371041686

[16] Joshi M, Farooq U, Mehrok S, Srouji N. Delayed formation of pulmonary artery stump thrombus: a case report and review of the literature. Thrombosis Journal 2009;7:7 doi: 10.1186/1477-9560-7-7 19515226 10.1186/1477-9560-7-7 PMC2699334

[17] Lazopoulos A, Asteriou C, Rallis T, Barbetakis N. Late postpneumonectomy death from stump thrombus. Ann Thorac Surg 2015;100(1): 330 doi: 10.1016/j.athoracsur.2015.03.048 26140787 10.1016/j.athoracsur.2015.03.048

[18] Kilic D, Akins S, Findikcioglu A, Bilen A, Aribogan A, Hatipoglu A. Low-molecular-weight heparin for treatment of submassive pulmonary embolism after pneumonectomy. Gen Thorac Cardiovasc Surg 2008;55(7):287–9. doi: 10.1007/s11748-007-0124-8 17679257 10.1007/s11748-007-0124-8

[19] Steriff MB. The National Comprehensive Cancer Network (NCCN) guidelines on the management of venous thromboembolism in cancer patients. Thromb Res 2010;125 Suppl2: S128–S33. doi: 10.1016/S0049-3848(10)70030-X 20433992 10.1016/S0049-3848(10)70030-X

[20] Agzarian J, Hanna WC, Schneider L, Schieman C, Finley CJ, Peysakhovich Y, et al Postdischarge venous thromboembolic complications following pulmonary oncologic resection: an underdetected problem. J Thorac Cardiovasc Surg 2016;151(4):992–9. doi: 10.1016/j.jtcvs.2015.11.038 26707765 10.1016/j.jtcvs.2015.11.038

